# THC in breath aerosols collected with an impaction filter device before and after legal-market product inhalation—a pilot study

**DOI:** 10.1088/1752-7163/acd410

**Published:** 2023-05-22

**Authors:** Kavita M Jeerage, Cheryle N Beuning, Adam J Friss, L Cinnamon Bidwell, Tara M Lovestead

**Affiliations:** 1 Applied Chemicals and Materials Division, National Institute of Standards and Technology, 325 Broadway, Boulder, CO, United States of America; 2 Department of Psychology and Neuroscience, University of Colorado, Boulder, CO, United States of America; 3 Institute of Cognitive Science, University of Colorado, Boulder, CO, United States of America

**Keywords:** exhaled breath aerosols (EBAs), cannabinoids, cannabis breathalyzer, marijuana, naturalistic, delta-9-tetrahydrocannabinol (THC)

## Abstract

An accurate cannabis breathalyzer based on quantitation of the psychoactive cannabinoid Δ^9^-tetrahydrocannabinol (THC) could be an important tool for deterring impaired driving. Such a device does not exist. Simply translating what is known about alcohol breathalyzers is insufficient because ethanol is detected as a vapor. THC has extremely low volatility and is hypothesized to be carried in breath by aerosol particles formed from lung surfactant. Exhaled breath aerosols can be recovered from electrostatic filter devices, but consistent quantitative results across multiple studies have not been demonstrated. We used a simple-to-use impaction filter device to collect breath aerosols from participants before and after they smoked a legal market cannabis flower containing ∼25% Δ^9^-tetrahydrocannabinolic acid. Breath collection occurred at an intake session (baseline-intake) and four weeks later in a federally-compliant mobile laboratory 15 min before (baseline-experimental) and 1 h after cannabis use (post-use). Cannabis use was in the participant’s residence. Participants were asked to follow a breathing maneuver designed to increase aerosol production. Breath extracts were analyzed by liquid chromatography with tandem mass spectrometry with multiple reaction monitoring of two transitions for analytes and their deuterated internal standards. Over more than 1 yr, 42 breath samples from 18 participants were collected and analyzed in six batches. THC was quantified in 31% of baseline-intake, 36% of baseline-experimental, and 80% of 1 h post-use breath extracts. The quantities observed 1 h post-use are compared to those reported in six other pilot studies that sampled breath at known intervals following cannabis use and are discussed with respect to participant characteristics and breath sampling protocols. Larger studies with verified abstinence and more post-use timepoints are necessary to generate statistically significant data to develop meaningful cannabis breathalyzer technology.

## 1. Introduction

Decriminalization and legalization of cannabis in many countries (e.g. Canada in 2018) and across most of the United States has coincided with a surge in medical and recreational use and concern regarding impaired driving skills. Cannabis impairs executive function [1] and in occasional users, cannabis increases the standard deviation of lateral position during simulated drives, a measure that indicates the extent of weaving within a lane [2, 3]. Composite drive scores from simulated drives were significantly worse at both 30 min and 1 h 30 min following cannabis use [4]. At 30 min, approximately half of the participants (more than 100 in total) stated they would drive in their current state, while at 1 h 30 min, the fraction increased to two-thirds, despite their measured impairment. Δ^9^-tetrahydrocannabinol (THC), the primary psychoactive molecule in cannabis, is predominantly found in the plant as Δ^9^-tetrahydrocannabinolic acid (THC-A) and is generated by decarboxylation during heating. THC-dominant recreational cannabis (>15% THC-A) comprises over 70% of the total product available in nine states, including Colorado, Washington, and California [5]. THC interacts with the nervous system through the endocannabinoid system, specifically, the cannabinoid receptors CB1 (abundant in the central nervous system) and CB2 (abundant in the immune system) [6]. As a deterrent to cannabis-impaired driving, some states have defined *per se* blood limits for THC, while others have adopted zero-tolerance policies for THC or its metabolites: the psychoactive 11-hydroxy-Δ^9^-THC (THC-OH) and/or the non-psychoactive 11-nor-9-carboxy-Δ^9^-THC (THC-COOH) [7]. While whole blood THC concentrations above 5 ng ml^−1^ have been associated with driving deficits in occasional cannabis users [2, 3], THC concentration in blood has not been consistently correlated to driver impairment [8]. THC is lipophilic and has limited solubility in blood, which means it can be stored in fatty tissue, resulting in prolonged and non-uniform release into blood. For daily users who resided on a closed research unit, THC remained detectable in blood for days and even weeks after cannabis use [9].

Blood sampling is also invasive. While urine sampling is non-invasive and is widely used to screen for cannabis use in the workplace, THC-COOH can be detected in urine for days or months, depending on frequency of use. Oral fluid sampling is non-invasive, observable, and is already used by law enforcement to confirm drug use in some countries. When smoked or vaporized, THC rapidly contaminates oral mucosa, leading to oral fluid concentrations of 1 *µ*g ml^−1^–2 *µ*g ml^−1^ 1 h after cannabis use [10–12]. THC concentration in oral fluid is, again, not consistently correlated to driver impairment [8] and oral fluid samples may be THC-positive 72 h after cannabis use [12]. While each of these biological matrices has strengths and limitations, methods employing non-invasive matrices to detect *recent use* remain an urgent need.

Breath sampling is noninvasive, difficult to adulterate, and widely accepted by law enforcement to determine alcohol impairment at the roadside. THC was first recovered from breath samples in the 1970s; with the low sensitivity methods available at that time, THC was detected for approximately 10 min following use [13]. THC and other cannabinoids are not like ethanol. They are large molecules with extremely low volatility [14] and are therefore hypothesized to be carried in exhaled breath aerosols, which are endogenously generated particles that form from respiratory tract lining fluid, a lipophilic lung surfactant [15]. Breath aerosols can be recovered from exhaled breath condensate (EBC) which contains water, volatile compounds, and aerosols. For example, when opioid drugs are delivered directly into the bloodstream, metabolites have been detected and quantified, e.g. normorphine from patients treated with morphine and dihydromorphine from patients treated with hydromorphone [16]. This result demonstrates the potential for breath aerosol analysis to detect systemic drugs. For inhaled drugs, residual material in the lungs may also contribute. To our knowledge, EBC samples have not been analyzed for cannabinoids.

Breath aerosols can also be recovered from filtration materials. The first devices utilized Empore solid-phase extraction disks, which contain C_18_ bonded silica sorbents within a polytetrafluoroethylene matrix and required a membrane pump to force breath through the filter [17, 18]. Electrostatic filters (ExaBreath device by SensaSure Technologies, formerly SensAbues) [19–23] and a combination filter containing a packed bed of silica particles plus an electrostatic filter (Hound Labs device) [24] have been used in subsequent studies with cannabis users. Breath aerosols are extracted from these devices with methanol (and pressure) and the extract is prepared for analysis by liquid chromatography with tandem mass spectrometry (LC-MS/MS) to identify and quantitate drugs. One known challenge is that solvent retention impacts cannabinoid recovery; the electrostatic filter absorbs approximately 3 ml methanol [19]. This may contribute to low cannabinoid recovery, which was first investigated by Himes *et al*, and certainly contributes to the complexity of the extraction procedure [19, 22].

Breath aerosol collection with electrostatic filters has been implemented in settings where the participants’ drug-use history was obtained by interview or was unknown [25–27]. For example, THC was detected in the breath of approximately half of participants who were positive for cannabis by blood, serum, or urine analysis [27]. Participants reported preferring breath sampling to blood or urine collections [26]. These studies support the idea that breath aerosol collection is straightforward for police personnel to implement. Himes *et al* conducted the first highly controlled study, in which participants resided in a closed research unit for 16 h to 20 h prior to cannabis use [19]. Subsequent studies in which participants were monitored for 3 h to 4 h following cannabis use demonstrated that THC in breath increases immediately after cannabis use [20, 22], decreases with time [19–24], and, importantly, that daily cannabis users may have THC in their breath despite self-reported abstinence for 12 h to 24 h [23, 24]. THC has been detected in breath samples collected approximately 24 h after admission to an inpatient treatment clinic with verified abstinence, which further supports this finding [25].

**Figure 1.**
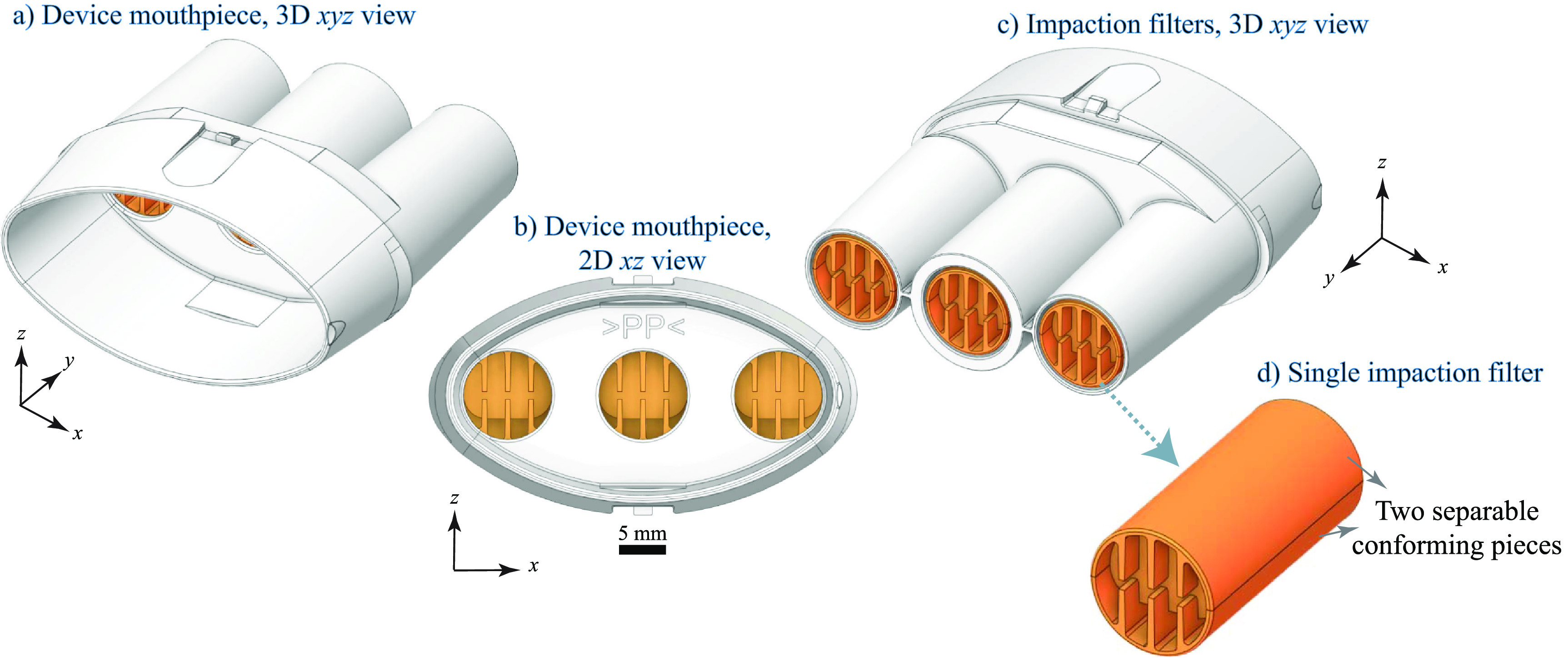
BreathExplor impaction filter device contains a mouthpiece (a), (b) and three impaction filters in parallel (c), which can be removed for elution (d). The impaction filters are shown with consistent orientation in (b), (c), but are oriented randomly in real devices.

Although the electrostatic filter (ExaBreath) device provides an easy-to-use method for breath aerosol collection that has been investigated since 2011, standardized protocols have not yet been adopted, based on the pilot-scale studies conducted to date. We examined the use of a newer impaction filter device (BreathExplor) that utilizes eight alternating baffles to direct fluid flow and to promote capture of breath aerosols. The overall device (figure 1) consists of a small, injection-molded medical grade polypropylene plastic tube with a mouthpiece (figures 1(a) and (b)) and three separate and parallel impaction filters (figures 1(c) and (d)). If the three identical filters provide the same results for breath aerosol composition, they could be analyzed separately for roadside detection and laboratory confirmation, for example, or for archival purposes. Limited studies to date indicate that the quantity of the lung surfactant dipalmitoyl phosphatidylcholine recovered via the central vs. the side filters was not significantly different [28]. Methadone was consistently recovered from patients on methadone maintenance [28] and illicit drugs, primarily cocaine and amphetamine, were detected in 13% of a large population of more than 1000 nightlife attendees [29]. Interestingly, THC was not detected in the 29 breath samples obtained from participants who self-reported recent cannabis use, though THC was detected in 9 other breath samples from this population [29], supporting the need for studies with known post-use timepoints.

This pilot study investigates the potential of a simple impaction filter device for breath aerosol collection that appears to offer advantages over electret filter devices and bridges the gap between highly controlled clinical studies and field studies that do not control for the time since cannabis use. Participants used a single, legal-market THC-dominant cannabis flower. They provided two baseline breath samples on different days and one post-use breath sample approximately 1 h after cannabis use, which is within the impairment window for driving deficits identified in simulator studies [4]. We end with recommendations for future studies based on our results and those of previous pilot studies to provide a scientific foundation for meaningful and reliable cannabis breathalyzer technology.

## 2. Naturalistic cannabis administration

Breath samples were collected from November 2020 to May 2022 in conjunction with a longitudinal study of cannabis use and anxiety (Novel Approaches to Understanding the Role of Cannabinoids and Inflammation in Anxiety, NIDA R01DA044131, CU IRB No. 16–0767) at the University of Colorado Center for Health and Neuroscience, Genes, and Environment. The study design allows participants to familiarize themselves with a specific product for four weeks between the intake and the experimental sessions. Participants were asked not to use cannabis the day before both the intake session and the experimental session. They were also asked to avoid using caffeine and tobacco products for 4 h before their session and informed that they had to pass an alcohol breathalyzer test with a reading of 0.00 to participate.

### 2.1. Intake session

Participants within the THC-dominant cannabis flower group were invited to participate in the pilot breath collection study (Chemical Foundations for a Cannabis Breathalyzer, DJO-NIJ-19-0008, NIST IRB No. MML-2019-0182); not all individuals chose to participate. After the larger study’s cognitive and behavioral assessments were completed, participants were instructed on a breathing maneuver designed to increase breath aerosol production. Participants provided a baseline breath sample following the maneuver for 12 exhalations. Participants were instructed to purchase a specific THC-dominant cannabis flower product sold by a licensed dispensary to use *ad libitum* until the scheduled experimental session four weeks later. Cannabinoid concentrations in the study product were measured periodically by an accredited lab: approximately 25% THC-A, 1.5% THC, <1% cannabidiolic acid and cannabidiol (CBD), <1% cannabigerolic acid, and 0% cannabigerol, cannabinol (CBN), and cannabichromene.

### 2.2. Experimental session

A federally-compliant mobile laboratory designed for evaluating the effects of legal-market cannabis use met participants at their residence [30]. After the larger study’s assessments were completed, which included blood collection, the baseline breath sample was collected. Participants then returned to their residence to use cannabis, *ad libitum* and unobserved by researchers (i.e. naturalistic use). Once participants returned to the mobile laboratory, the larger study’s assessments were completed, which included immediately collecting a blood sample. These assessments took approximately 1 h. Finally, the post-use breath sample was collected. Procedures for venous blood collection and plasma analysis have been described [30].

## 3. Breath aerosol sample collection

BreathExplor components including devices, filters (within the devices), filter transfer tools, and 2 ml elution vials were made from medical-grade polypropylene and were provided in kind by Munkplast AB, Inc. Devices were kept in the mobile laboratory, remained sealed until use, and were never in the same place that cannabis was consumed. Participants were asked to exhale through the device 12 times following a specific breathing maneuver: (1) fully exhale until they reached their residual volume, (2) hold their breath for 10 s, (3) inhale until they reached their total lung capacity, (4) place the device into their mouth, and (5) exhale until they reached their functional residual capacity. Research with non-impaired participants has shown that full exhalation increases the formation of aerosols by allowing the airways to close [31, 32]. Low-lung-volume breath holds have a similar, but smaller effect on aerosol production [33]. Devices were recapped, sealed in a plastic bag, stored in a cooler while in transit, and stored at −80 °C at the University of Colorado. The devices were transferred to the National Institute of Standards and Technology (NIST) where they were stored at −20 °C until analysis in small batches. Baseline breath samples collected at the intake session were stored for at least four weeks to allow for the complete set of samples from each participant (if available) to be processed together. Samples were also stored such that each batch contained six to eight breath extracts. Due to gaps in recruitment outside our control and pandemic-related restrictions, some breath samples were stored for 30 weeks.

## 4. Analyte extraction and concentration

### 4.1. Chemicals

Certified reference materials for analytes, THC, CBD, CBN, THC-OH, and THC-COOH, and their deuterated internal standards (denoted by -d3) were purchased as ampules, used as received, and had reported purities from 98.8% to 99.9%. LCMS-grade methanol, water, and formic acid were used as received. Ethylene glycol had a purity of ⩾99%. All solutions were prepared gravimetrically in clear silanized glass vials. Stock solutions were stored at −20 °C and were used within 60 d. All dilutions of stock solutions were prepared within 48 h of analyses and stored at −20 °C until analyzed by LC-MS/MS.

### 4.2. Device processing

We analyzed breath extracts in six batches (I through VI). To prepare these extracts, devices were first warmed to ambient temperature. Filters were removed from the housing (figures 1(a) and (b)) using the manufacturer provided tool to push them from the mouthpiece. Analyte extraction was from the filters only, not the mouthpiece or the portion of the device that houses the filters. Each filter was submerged and soaked separately for 10 min–15 min in 1.5 ml of methanol containing ethylene glycol, which was added to the elution solvent to retain analytes during concentration based on manufacturer recommendation. Filters were removed from the eluent and centrifuged to recover residual eluent. The combined eluent (from all three filters) was spiked with an internal standard solution and dried with a vacuum concentrator at 35 °C for 150 min. The resulting pellet, primarily ethylene glycol containing analytes, was solvated with 100 *µ*l 30% water/70% methanol for analysis by LC-MS/MS. Calibration standards were prepared in methanol with ethylene glycol (matrix-matched) and were dried and reconstituted as described above (process-matched). Five quality control (QC) samples were created and analyzed alongside each batch of breath extracts. The final concentration of ethylene glycol varied by batch; calibration standards had average concentrations that ranged from 5.3% to 7.8% in the reconstitution solvent. Breath extracts had average concentrations that ranged from 5.2% to 8.1% and were more variable due to soaking the filters, which led to differences in solvent loss during the elution process. Similarly, the final concentration of internal standard varied by batch due to differences in the concentration of the internal standard spike solution; calibration standards had THC-d3 concentrations that ranged from 11.0 ng g^−1^ (Batch VI) to 18.3 ng g^−1^ (Batch IV). Differences in solvent loss during the elution process and solvent evaporation during the vacuum concentration process led to THC-d3 concentrations for five extracts that varied by 20% or more from the calibrators for that batch.

### 4.3. Elution efficiency

Breath matrix was added to devices by a non-cannabis user following the prescribed breathing maneuver. Filters were removed and condensed water was allowed to evaporate at room temperature for 16 h (overnight). Individual filters were then spiked with THC in ethanol (20 *µ*l aliquots) and the solvent was allowed to evaporate at room temperature for 3 h. Filters were immediately eluted, as described above, or stored at −20 °C and eluted periodically. THC spikes for immediately eluted filters (18) were less than 1 ng/filter. THC spikes for stored filters (nine per storage period) were increased to 2.5 ng/filter. Eluents were not combined; each filter was individually analyzed. After eluents and calibration standards were dried with the vacuum concentrator, the resulting pellets were solvated with 30% water/70% methanol for analysis by LC-MS/MS. In these experiments, the reconstitution solvent also contained THC-d3 and CBN-d3 internal standards, yielding THC-d3 concentrations with a coefficient of variance of less than 2%.

## 5. LC-MS/MS instrumentation and parameters

Cannabinoids were separated on an Agilent InfinityLab Poroshell 120 EC-C18 reversed-phase column (100 mm length, 2.7 *µ*m particle diameter) preceded by a 5 mm guard column on an Agilent Infinity 1290 ultra-high-pressure LC instrument. Cannabinoids were detected with an Agilent 6460 or 6470 triple quadrupole tandem MS instrument in positive polarity electrospray ionization mode (table S1). Agilent Masshunter and Optimizer software packages were used to determine the most abundant quantifier, *Q*, and qualifier, *q*, product ions for each precursor ion and their respective collision and fragmentor energies from standard solutions (table S2). Figure S1 illustrates the chromatographic separation of the five analytes studied here.

### 5.1. Cannabinoid identification and quantitation

Positive identification of a compound in a breath extract required, first, that the analyte’s retention time was within ±0.3 min of its expected retention time based on calibration standards and within 0.05 min of its deuterated internal standard, and second, that its product ion ratio (*q*/*Q*) was within ±20% of the ratio calculated for its calibration standard and its internal standard. Potential contamination was investigated by analyzing solvent blanks without and with internal standards and by extracting and concentrating analytes from an unused device. Potential interference from breath compounds not originating from cannabis was examined by extracting and concentrating analytes from a breath sample generated by a non-cannabis user. Solvent blanks were also used to rule out cannabinoid carryover by injecting the highest calibration standard and then injecting a solvent blank.

Calibration standards were prepared to include both a high and low analyte concentration range, including concentrations expected to be below the limit of detection (LOD). Linear regression with a 1/*x* weighting function was used for all calibration curves. Calibration standards with signals indistinguishable from noise were removed and regression analysis with a concentration range spanning at least three orders of magnitude was used to guide identification of the calibration standards used for the LOD (LOD = *S*/*N* ⩾ 3) and the limit of quantitation (LOQ = *S*/*N* ⩾ 10). Calibration standards were then used to generate two calibration curves for analyte quantitation. QC 1 and QC 2 were quantified with the high calibration range and QCs 3–5 were quantified with the low calibration range. Calibration curve coefficients of determination (*R* ^2^) were ⩾0.99 for each analyte.

## 6. Results

Table 1 provides LODs and LOQs for THC; results for the remaining analytes are also provided (tables S3–S6). LODs for THC, CBD, and CBN ranged from 0.004 ng/device to 0.05 ng/device, depending on the batch. LODs for THC-OH and THC-COOH were higher and ranged from 0.008 ng/device to 0.08 ng/device. THC was identified in 31% of baseline-intake, 36% of baseline-experimental, and 80% of post-use breath extracts. CBD was identified in three breath extracts and CBN in five breath extracts. THC-OH and THC-COOH were not detected in any breath extracts. Tables 1 and S3–S6 show that quantitative accuracies for Batch I were outside typical acceptance limits. Unfortunately, in this batch, the internal standard added to the QC samples was 20%–30% lower than the corresponding calibration standards, leading to high relative responses and calculated concentrations. Internal standard added to the breath extracts was not affected.

**Table 1.** THC limit of detection (LOD) and limit of quantitation (LOQ) over the course of the study. Quantitative accuracy for the quality control (QC) samples was calculated by the equation: ${\text{Accuracy}}\left( \% \right) = 100 - 100 \times \left( {\left( {{V_{\text{T}}} - {V_{\text{O}}}} \right)/{V_{\text{E}}}} \right)$ where ${V_{\text{T}}}$ is the true value calculated by gravimetry and ${V_{\text{O}}}$ is the observed value calculated by the calibration curve. THC quantities are reported in 30% water and 70% methanol with ethylene glycol. The ‘n/a’ indicates that the QC concentration is below LOQ. Gravimetric QC concentration ranges by batch: I (82 ng g^−1^–0.1 ng g^−1^), II (144 ng g^−1^–0.4 ng g^−1^), III (149 ng g^−1^–0.6 ng g^−1^), IV (170 ng g^−1^–0.6 ng g^−1^), V (153 ng g^−1^–0.07 ng g^−1^) and VI (102 ng g^−1^–0.05 ng g^−1^).

Series/Date	I	II	III	IV	V	VI
LOD (ng/device)	0.01	0.01	0.03	0.05	0.004	0.02
LOQ (ng/device)	0.02	0.03	0.04	0.08	0.007	0.02
LOD (ng g^−1^)	0.1	0.1	0.3	0.6	0.05	0.1
LOQ (ng g^−1^)	0.2	0.3	0.4	0.8	0.07	0.2
Quantitative accuracy (%)						
QC 1 (82–170 ng g^−1^)	130	110	110	110	96	110
QC 2 (11–32 ng g^−1^)	130	110	100	92	92	97
QC 3 (1.0–2.7 ng g^−1^)	120	97	95	99	93	100
QC 4 (0.1–1.3 ng g^−1^)	120	94	110	98	100	n/a
QC 5 (0.05–0.6 ng g^−1^)	n/a	99	110	n/a	n/a	n/a

Table 2 provides quantitative values for THC, CBD, and CBN. With one exception (I-1), breath extracts were quantified with the low calibration range. THC in post-use extract I-1 was 40× more than the next highest extract. Of the 14 participants who provided two samples during the experimental session, eight participants showed the anticipated increase in THC after cannabis use. THC was not detected in three post-use breath extracts and the remainder of post-use extracts were similar to or lower than baseline extracts. THC quantities, when detected and with one exception (I-1), were similar in baseline and post-use extracts. While carryover was never seen, a potential interferent was observed in two filter blanks (Batches V and VI). However, this interferent was not observed in the breath or solvent blanks. THC was quantified in 7 of the 13 extracts analyzed in these batches and are reported here without attempting to correct the signal for the interferent.

**Table 2.** THC (light green shading), CBD (no shading), and CBN (light yellow shading) reported in ng/device based on the average of four injections. Gray shading indicates that the participant did not provide a breath sample. Trace, *tr*, indicates values above the LOD but below the LOQ. Dashes indicate that the analyte was not detected.

	Intake sessions			Experimental sessions (4 weeks later)					
	BASELINE			BASELINE			POST-USE (1 h)		
ID	THC	CBD	CBN	THC	CBD	CBN	THC	CBD	CBN
I-1	—	—	—	—	—	—	21	0.03	0.5
I-2	—	—	—	—	—	—	—	—	—
II-1	—	—	—	0.5	*tr*	*tr*	0.2	—	—
II-2				0.2	—	—	0.2	—	—
II-3	0.05	—	—	0.2	—	—	0.5	—	*tr*
III-1	—	—	—				0.04	—	.—
III-2				0.2	—	—	—	—	—
III-3	—	—	—	0.1	0.9	—	0.06	—	—
IV-1				—	—	—	0.2	—	0.09
IV-2				—	—	—	—	—	—
IV-3	—	—	—						
IV-4	—	—	—						
IV-5				—	—	—	0.5	—	—
V-1	—	—	—	—	—	—	0.1	—	—
V-2	—	—	—	—	—	—	0.07	—	—
VI-1	0.04	—	*tr*						
VI-2	0.04	—	—	—	—	—	0.1	—	—
VI-3	0.3	—	—	—	—	—	0.04	—	—

It appears that baseline extracts collected at intake sessions had less THC than baseline extracts collected at experimental sessions. This may be a consequence of the recruitment criteria and the study design, i.e. in order to enroll in the study, participants were required to have ‘prior experience with cannabis’ at no specific frequency or recency and were interested in starting to use cannabis to relieve anxiety. At the intake session, participants self-reported cannabis use events for the previous 14 d. Six participants reported 0 d, while three participants reported 13 d or more. The remainder reported 2 d–9 d of cannabis use prior to the intake session. Therefore, the four week study period captures an intended uptick in cannabis use.

One challenge in a naturalistic study design is that cannabis use is unobserved; therefore, the larger study’s protocol includes measuring compliance indirectly by sampling venous blood before and *directly after* cannabis use, i.e. as soon as the participant returned to the mobile laboratory. Participants included here spent an average of 16 min away from the mobile laboratory (range from 6 min to 29 min). Table 3 presents their blood data binned into five groups. Twelve participants had the expected increase in THC plasma concentration immediately after cannabis use, and half these participants had THC plasma concentrations greater than 50 ng ml^−1^. Surprisingly, three participants had no detectable THC in their blood immediately after cannabis use and two participants had THC plasma concentrations less than 10 ng ml^−1^. In a naturalistic study of high-potency cannabis flower and concentrates (*N* = 133), Bidwell *et al* excluded 12 participants due to low post-use THC plasma concentrations (<20 ng ml^−1^ vs. the study mean of 240 ng ml^−1^) [30]. Altogether, the results in table 3 may indicate that three participants did not smoke cannabis in their home (i.e. did not comply with the protocol) or that their typical cannabis use does not result in detectable THC in blood plasma. Note that blood data are only used here as an indication of compliance with the protocol—no participants were excluded. In a real-world scenario, it is unrealistic to obtain a blood sample immediately following cannabis use.

**Table 3.** THC plasma concentrations in ng ml^−1^ (*n*) measured from blood collected immediately after cannabis use, *directly after* returning to the mobile laboratory. THC concentrations are binned into five groups: (1) below the limit of quantitation (BLOQ), (2) above the LOQ but below 1 ng ml^−1^ (*n*< 1), (3) above 1 ng ml^−1^ but below 10 ng ml^−1^ (1 < *n*< 10), (4) above 10 ng ml^−1^ but below 50 ng ml^−1^ (10 < *n*< 50), and (5) greater than 50 ng ml^−1^ (*n* > 50). Blank fields indicate that no data was available for that participant and session.

	Intake sessions	Experimental sessions (4 weeks later)	
ID	BASELINE	BASELINE	POST-USE (immediate)
I-1	1 < *n* < 10	1 < *n* < 10	*n* > 50
I-2	*n* < 1	1 < *n* < 10	*n* > 50
II-1	BLOQ	BLOQ	10 < *n* < 50
II-2	1 < *n* < 10	1 < *n* < 10	*n* > 50
II-3	10 < *n* < 50	*n* > 50	*n* > 50
III-1	BLOQ		
III-2	BLOQ	BLOQ	BLOQ
III-3	BLOQ	BLOQ	10 < *n* < 50
IV-1	1 < *n* < 10	1 < *n* < 10	10 < *n* < 50
IV-2	BLOQ	BLOQ	1 < *n* < 10
IV-3	BLOQ		
IV-4			
IV-5	1 < *n* < 10	1 < *n* < 10	*n* > 50
V-1	BLOQ	BLOQ	1 < *n* < 10
V-2	BLOQ	BLOQ	10 < *n* < 50
VI-1	BLOQ	1 < *n* < 10	BLOQ
VI-2	*n* < 1	1 < *n* < 10	*n* > 50
VI-3	BLOQ	BLOQ	BLOQ

## 7. Discussion

### 7.1. Study design and procedures

Breath sampling in the mobile laboratory following cannabis use has many of the strengths and limitations that might be experienced during roadside breath sampling. For example, the BreathExplor impaction filter devices were never in the same location where cannabis was consumed, because naturalistic use [30] of a legal-market product [34] occurred within each participant’s residence. However, cannabis use was unobserved and the time interval from use to breath sampling has greater uncertainty than studies conducted in controlled clinical environments. Ambient temperature during breath sampling also varied, as samples were collected year-round in Colorado, including one month between intake and experimental sessions. Participants were observed during breath sampling and their exhalations through the device were counted. Our original protocol also included equipping the devices with a spirometer to measure breath volume and flow rate; breath volume is an important criterion to ensure a valid sample for the alcohol breathalyzer. Unfortunately, assembling these components and manipulating the spirometry software to measure each exhalation required close contact between participants and researchers. Therefore, spirometry was ultimately excluded to allow the study to proceed during the COVID-19 pandemic.

While breath researchers designed this portion of the study and trained the research staff interacting with participants, they could not be involved in breath sampling or interact with participants. The research staff reported that some participants found the low-lung-volume breathing maneuver, implemented to increase the production of breath aerosols, uncomfortable. They also reported that participants interspersed normal breathing (not through the device) with the breathing maneuver and, therefore, participants took approximately 10 min to complete 12 exhalations through the device. Some participants only completed ten exhalations. Based on previous studies, deep breaths appear to have a greater effect on aerosol production than low-lung-volume breath holds [32, 33]. Therefore, with a small number of participants not otherwise included here, we modified the breathing maneuver to require a 3 s low-lung-volume breath hold rather than a 10 s breath hold. This appears to reduce discomfort such that all exhalations are through the device, and approximately 25 exhalations can be sampled in 5 min (data not shown).

Potential contamination with oral fluid is a concern for all breath sampling based on the high THC concentration found in oral fluid when cannabis is smoked or vaped. Oral fluid contamination could be assessed by extracting and analyzing for alpha-amylase (if present) [35], but we did not do that here because extracts from all three filters were combined to maximize cannabinoid content in the final extract. We made this choice based on analysis of individual filters from one participant (data not shown). Future studies, including empirical and modeling studies are necessary to investigate this important question.

Analyte extraction from an impaction filter appears straightforward compared to an electrostatic filter that retains solvent. Residual solvent trapped within the filter was recovered by brief centrifugation and total solvent loss (transfer loss *and* evaporative loss) was less than 10% by volume. While loss during transfer (to pipet tips etc) results in loss of analyte, evaporative loss is assumed not to be a problem based on the low vapor pressure of cannabinoids [14]. However, these losses cannot be distinguished. We added internal standard to the combined eluent after filter removal; this does not account for cannabinoids (if any) retained by the filter. We made this choice because spiking the impaction filter with 40 *µ*l aliquots of internal standard in methanol leads to solution pooling in the vial. Thus, the captured analytes and their spiked internal standards may experience different forces during elution. THC elution efficiency was investigated here with individual impaction filters containing dried breath matrix; 20 *µ*l aliquots of analyte in ethanol were used to spike the filter surfaces and minimize solution pooling. These experiments suggest that despite good recovery of the elution solvent (approximately 90%), THC recovery is low. Filters eluted immediately after the aliquot dried had average recoveries of 23 (±5) %. When filters were stored at −20 °C, average recoveries decreased further. Three storage periods have been investigated to date. Recoveries were 18 (±6) % after two weeks. Electrostatic filters also have known challenges, such as analyte loss due to adsorption and solvent retention and low (34%) THC recovery [19]. Analyte extraction and concentration has not been fully standardized and reported LOQs in two recent studies include 0.01 ng/device [22] and 0.2 ng/device [23]. Future analyte extraction studies are needed to understand and optimize cannabinoid recovery.

### 7.2. Results in context of peer-reviewed literature

To date (March 2023), six peer-reviewed studies have been published in which breath aerosols were collected with filter-based devices at known intervals following cannabis use [19–24]. Table 4 summarizes some aspects of these studies. We requested one day of abstinence (unverified) and sampled baseline concentrations at two separate sessions. In other studies, baseline concentrations were sampled at a single session.

**Table 4.** Instructions with respect to abstinence, breath sampling protocol indicated by time, exhaled breaths, and/or volume as presented in the original publications, and timepoint closest to 1 h.

Author-year	Instructions prior to experimental session	Sampling protocol			Post-use time (h)
		Time	Breaths	Volume	
Himes *et al* 2013 [19]	Abstinence requested and verified (16 h–20 h).	3 min			0.7–1.1
Coucke *et al* 2016 [20]	Abstinence not requested.	2–3 min		30 l	1.0
Kintz *et al* 2017 [21]	Abstinence not requested.		20		1.0
Lynch *et al* 2019 [24]	Abstinence requested (24 h) but not verified.			18 l	1.0
Olla *et al* 2020 [22]	Abstinence not requested.		25		1.5
Wurz and DeGregorio 2022 [23]	Abstinence requested (12 h) but not verified.^a^	2–3 min		20 l	1.0

^a^ Wurz *et al* also specified that participants use cannabis between 12 h and 24 h prior to their scheduled experimental session.

Table 2 indicates that we detected THC in 33% of baseline breath extracts. While Lynch *et al* detected THC in all participants at baseline [24], this finding was enabled by a derivatization method that increased LC-MS/MS ionization efficiency. LOQs were lower than all other pilot studies [36]. Lynch *et al* reported one baseline concentration of 0.06 ng/device, but most were below 0.01 ng/device and thus below our detection limit. Table 3 indicates that many of our participants did not have any detectable THC in their blood plasma at either baseline session (17 of 32). Additionally, most of the remaining participants had THC plasma concentrations below 10 ng ml^−1^ (13 of 32). Baseline concentrations in other studies may indicate different participant characteristics. For example, Olla *et al* reported an average THC plasma concentration of 16 ng ml^−1^ [22] while concentrations reported by Wurz *et al* correspond to an average THC plasma concentration of approximately 13 ng ml^−1^ [23, 37]. In our study, only one participant (II-3) had baseline THC plasma concentrations above 10 ng ml^−1^ and, indeed, THC was detected in all breath extracts from this participant.

**Figure 2.**
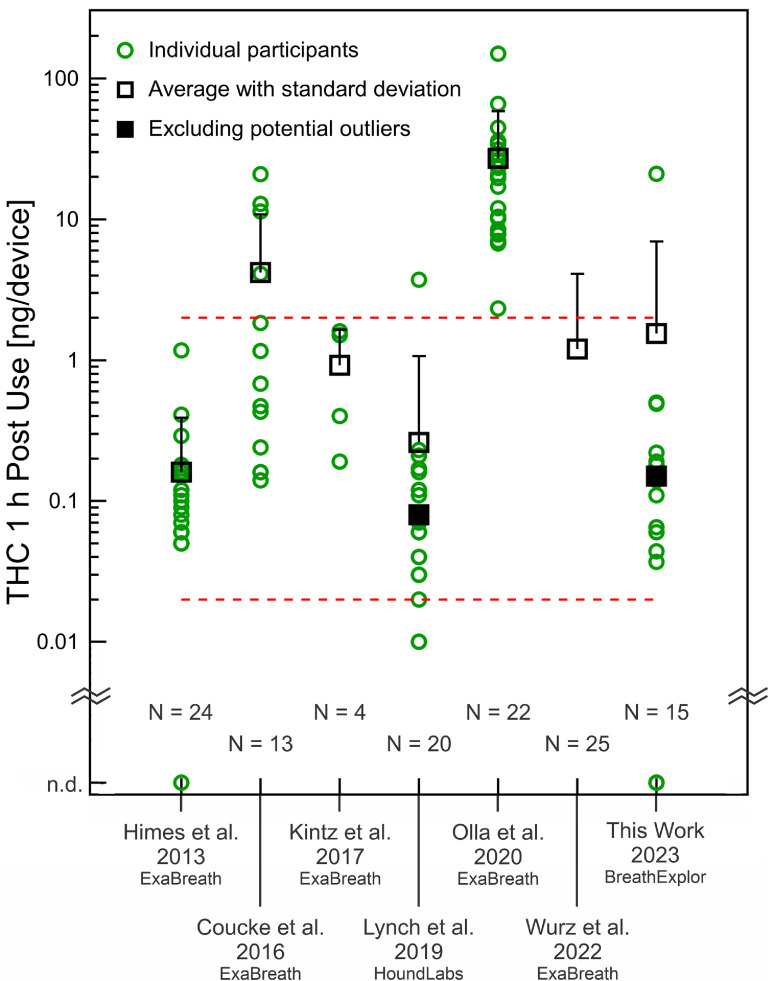
Comparison of THC (ng/device) recovered approximately 1 h after cannabis use with ExaBreath (electrostatic filter), HoundLabs (packed bed plus electrostatic filter), or BreathExplor (impaction filter) devices. Sample size (*N*) indicates the number of participants who completed this specific post-use timepoint, some studies had more participants, and all studies except ours had more post-use timepoints. Our post-use time was 1 h to 1.5 h. Wurz *et al* did not provide measurements for individual participants; the average and standard deviation provided here are based on figure digitization. Dashed red lines at 2 ng/device and 0.02 ng/device are to guide the eye. Himes *et al* had one participant with no THC in their post-use breath extract; we had three.

Figure 2 summarizes 1 h post-use measurements (or the closest timepoint) from the existing pilot studies (table 4), which primarily used the ExaBreath device (electrostatic filter); one used the HoundLabs device (packed bed plus electrostatic filter). Results from the first pilot-scale investigation of the BreathExplor impaction filter device (this work) are included for comparison. One hour after cannabis use, we measured THC in breath extracts at 1.5 ng/device (including participant I-1) and 0.15 ng/device without this participant, whose breath extract is a potential outlier. Lynch *et al* also identified a potential outlier and the averages for their data are calculated with and without this participant [24]. The participants studied by Himes *et al*, Coucke *et al*, and Lynch *et al* included some individuals with 0–2 d of use within the previous 14 d [19, 20, 24], similar to our participants. Figure 2 shows that approximately 1 h after cannabis use, most breath extracts from our participants and these three studies fell between 0.02 ng/device and 2 ng/device (dashed red lines). Participants studied by Olla *et al* stand out with multiple breath extracts an order of magnitude higher. Order of magnitude differences indicate a challenge for breathalyzer development.

One hour after cannabis use, our results with the new impaction filter device are broadly comparable to previous pilot studies, considering participant characteristics and breath sampling differences. However, we must also consider that THC in breath at 1 h post-use was not necessarily higher than baseline, even when THC in blood indicated compliance with the protocol and at least a five-fold increase immediately post-use (participants I-2 and II-2). This may be related to differences in breath sampling. Participants may have found the breathing maneuver even more challenging to execute when intoxicated or they may have been eager to complete the session—the post-use breath sample was the final procedure of the experimental session. Breathing differences could affect aerosol generation or aerosol capture by the filters. Further investigation is required to identify factors that lead to outliers based on sampling differences.

### 7.3. Recommendations for future studies

Averaged data from pilot studies with small numbers of participants can hide intra- and inter-individual variations and we appreciate that several of the publications discussed here made data available for each participant and timepoint sampled. Examining these datasets reveals additional examples where post-use breath extracts have less THC than baseline breath extracts; THC may also be unusually high or low in one breath extract [20, 22, 24]. These observations suggest that reproducible breath aerosol collection remains an ongoing challenge. We propose that spirometry measurements should be included in future studies, both to identify outliers based on sampling and to investigate whether factors such as flow rate play a role in breath aerosol capture. We also propose that THC-spiked aerosols generated in the laboratory would be a useful complement to human studies. If reproducible, such materials could be used to elucidate factors that influence elution efficiency and analyte recovery, compare different devices, and simulate different breathing patterns. Last, cannabis breathalyzer devices must be independently certified and standardized to lead to a useful device for forensics and public health and safety.

## 8. Conclusions

Since the first observation of THC in breath, THC has been detected in the breath of patients during general toxicology screens in which cannabis use was not the focus of the study design. The groundbreaking and highly controlled clinical study by Himes *et al* in 2013 suggested the potential for detecting recent cannabis use with a breath measurement. In the decade following, a handful of studies have successfully revealed the difficulties of developing a meaningful and reliable THC breath measurement for law enforcement. Put in the perspective of the alcohol breathalyzer, still undergoing developments to ensure accuracy after a 100 yr of fundamental and applied research, there is much to be investigated for reliable cannabis breathalyzer development. We have shown that a simple impaction filter device successfully collected breath aerosols from cannabis users, which were subsequently extracted, concentrated, and analyzed with laboratory instruments to quantify THC in baseline and 1 h post-use breath extracts. Quantitative values were broadly comparable to other pilot studies with different devices, sampling protocols, and participant characteristics. Our results do not support the idea that detecting THC in breath as a single measurement could reliably indicate recent cannabis use.

## Data Availability

All data that support the findings of this study are included within the article (and any supplementary files).
